# Tannic Acid-Directed Synthesis of Mesoporous Anatase TiO_2_: Linking Structural, Surface, and Thermodynamic Properties to Photocatalytic Performance

**DOI:** 10.1021/acs.langmuir.6c03788

**Published:** 2026-09-07

**Authors:** Stefani Pelloso de Azevedo Gobetti, Hugo Henrique Carline de Lima, Emerson Marcelo Girotto, Marcos Rogério Guilherme, Andrelson Wellington Rinaldi

**Affiliations:** Rinaldi Research Group, Department of Chemistry, 42487 State University of Maringá, 5790 Colombo Avenue, 87020-900 Maringá, PR, Brazil

## Abstract

Rational engineering of TiO_2_ surface architecture offers an effective approach to optimizing its physicochemical and photocatalytic properties in advanced oxidation processes (AOPs). Incorporation of organic structure-directing agents during synthesis provides a versatile strategy for tailoring the structural and surface properties of TiO_2_, although sustainable, naturally derived alternatives remain relatively unexplored. Tannic acid (TA), a low-cost and renewable polyphenolic compound, is particularly attractive for this purpose. Herein, a sustainable sol–gel approach was developed for the synthesis of mesoporous TiO_2_ using TA as a bio-derived organic structure-directing agent. Systematic variation of the TA concentration established a concentration-dependent structure–property–activity relationship linking the crystalline structure, morphology, surface characteristics, thermodynamic properties, and optical properties to photocatalytic performance under UV irradiation. The refined lattice parameters closely matched those of standard anatase TiO_2_, indicating that variations in TA concentration did not substantially alter the anatase crystal structure. Thermodynamic analysis of the oxidation pathways provided quantitative insight into the feasibility of hydroxyl radical formation. Methylene blue degradation followed apparent pseudo-first-order kinetics under the investigated conditions, with the photocatalytic activity reflecting the combined contributions of surface accessibility, light harvesting, and charge-carrier behavior. The results demonstrate that TA can effectively direct the formation of mesoporous TiO_2_, providing a sustainable route for tailoring photocatalyst properties and promoting pollutant degradation under low-energy UV-LED irradiation.

## Graphical abstract

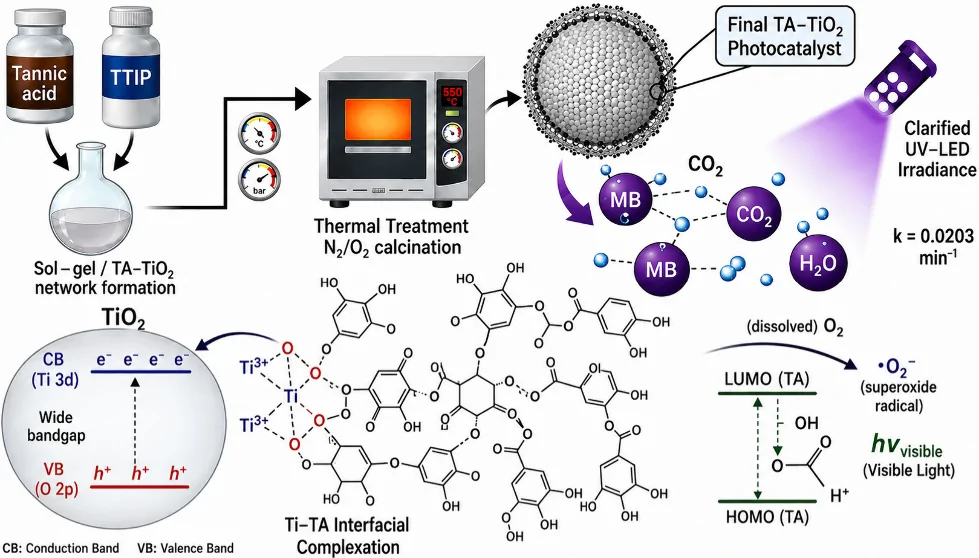

## Introduction

Organic dyes are important water contaminants, and their removal remains a significant environmental challenge.^1,2^ When inadequately treated, dye-containing effluents can severely impair aquatic ecosystems by deteriorating water quality, reducing light penetration, and exerting toxic effects on aquatic organisms. Advanced oxidation processes (AOPs) have therefore emerged as promising strategies for the degradation of recalcitrant organic contaminants. Among these technologies, photocatalysis has attracted considerable attention because its efficiency is strongly governed by processes occurring at the semiconductor–solution interface. Upon irradiation with photons possessing energy equal to or greater than the semiconductor band gap, electron–hole pairs are generated,^3^ followed by their migration toward the catalyst surface, where interfacial charge-transfer reactions can occur. At the surface, photogenerated charge carriers interact with adsorbed water, hydroxyl groups, dissolved oxygen, and organic molecules, promoting the formation of highly reactive species, including hydroxyl and superoxide radicals.^4^ The resulting surface-mediated oxidation processes promote the transformation of organic contaminants into intermediate species, which may undergo further oxidation toward less complex and potentially less harmful products. Accordingly, the physicochemical characteristics of the photocatalyst surface, including specific surface area, adsorption capacity, mesoporous structure, surface functional groups, and interfacial charge-transfer properties, play critical roles in determining photocatalytic activity.^,^ ^5^

Titanium dioxide (TiO_2_) is one of the most widely studied photocatalysts owing to its chemical stability, low cost, and favorable photocatalytic properties.^6^ However, its performance remains strongly dependent on structural and interfacial properties that govern light harvesting, charge-carrier dynamics, and surface reaction pathways.

The photocatalytic performance of TiO_2_ is governed by its ability to absorb light, produce and separate photogenerated charge carriers, and promote interfacial charge-transfer reactions. Beyond photocatalysis, TiO_2_ is attractive for biological applications owing to its chemical stability, low toxicity, and acceptable biocompatibility, which can vary with its composition and physicochemical properties.^7,8^ However, its relatively wide band-gap and rapid recombination of photogenerated electron–hole pairs can limit its photocatalytic efficiency. To address these limitations, various modification strategies have been explored, including hydrothermal,^9^ solvothermal,^10^ precipitation,^11^ mechanochemical,^12^ sol–gel approaches,^13^ and the use of structure-directing agents.^14^

Among these approaches, structure-directing agents enable control over the microstructural evolution of TiO_2_, thereby tuning its physicochemical properties. Complementary incorporation of metallic species, nonmetal dopants, and/or semiconductor components can further modulate light harvesting, charge-carrier dynamics, and surface reaction pathways.^,^ ^15,16^ Nonetheless, most conventional directing agents are petroleum-derived and their effects on structure–property relationships are not yet fully understood.^14,17^

The sol–gel technique has attracted particular interest because of its simplicity, versatility, and ability to produce homogeneous materials with tunable structural properties. In this approach, a colloidal suspension (sol) undergoes hydrolysis and condensation reactions, leading to the formation of a three-dimensional inorganic network (gel).^18^ To further tailor TiO_2_ architectures, the sol–gel process is frequently combined with soft-templating strategies employing structure-directing agents capable of regulating surface properties.^19^ Recent studies have demonstrated that organic acids such as citric,^20^ oxalic,^21^ picolinic,^22^ and glycolic acids^23^ can effectively act as structure-directing agents during TiO_2_ synthesis, resulting in enhanced photocatalytic activity.

Tannic acid (TA) represents a promising yet largely unexplored alternative for this purpose. As a naturally derived polyphenolic compound, TA combines low cost, renewable origin, and reduced environmental impact compared with conventional surfactants and synthetic templating agents.^24,25^ Moreover, its multiple phenolic hydroxyl groups can interact strongly with titanium precursors during sol–gel processing, potentially influencing nucleation, particle growth, and pore development.

Herein, a sol–gel strategy employing TA as a bio-based structure-directing agent was developed to enable the synthesis of TiO_2_ photocatalysts with tunable surface and physicochemical properties. By systematically varying the TA concentration, correlations were established between the synthesis conditions and the resulting crystalline structure, morphology, surface characteristics, optical properties, and photocatalytic activity under UV irradiation. To the best of our knowledge, this work represents the first demonstration of TA as a bio-based structure-directing agent for TiO_2_ synthesized via the sol–gel route.

## Materials and Methods

### Synthesis of TiO_2_ Using TA as a Structure-Directing Agent

TiO_2_ photocatalysts were synthesized according to a modified sol–gel procedure adapted from previous reports.^26^ TA (Sigma-Aldrich) was dissolved in 50.0 mL of ethanol under vigorous stirring using the amounts specified in Table 1. After complete dissolution, concentrated ammonium hydroxide was added, followed by the dropwise addition of titanium isopropoxide (TTIP, Sigma-Aldrich; 0.39 mL, 1.34 mmol). The resulting suspension was stirred continuously for 2 h to allow hydrolysis and condensation reactions

**Table 1:** Tannic Acid (TA) Content and TA:TTIP Molar Ratios Used in the Synthesis of TiO_2_ Photocatalysts

Photocatalysts	TA:TTIP molar ratio	TA mass (mg)	TA amount (mol)
TA0**-**TiO_2_	0.0:1.0	0.0	0.0
TA0.5**-**TiO_2_	0.5:1.0	8.50	5.0 × 10^–6^
TA1**-**TiO_2_	1.0:1.0	17.00	1.0 × 10^–5^
TA4**-**TiO_2_	4.0:1.0	68.00	4.0 × 10^–5^
TA16**-**TiO_2_	16.0:1.0	272.00	1.6 × 10^–4^

The resulting solids were recovered by centrifugation at 3400 rpm for 20 min, resuspended in ethanol for 24 h, dried at 100 °C, and stored in a desiccator prior to further use. For the development and crystallization of the TiO_2_ phases, the material was thermally treated in a muffle furnace (EDG3P-S 7000) under a nitrogen atmosphere at 450 °C for 2 h, using a heating rate of 1 °C min^–1^. Subsequently, the samples were maintained at 450 °C under a continuous oxygen flow for 30 min to remove residual.

The material yields, calculated from the recovered solid mass relative to the theoretical TiO_2_ yield, were 28.83%, 27.76%, 27.95%, 23.82%, and 17.05% for AT0–TiO_2_, AT0.5–TiO_2_, AT1–TiO_2_, AT4–TiO_2_, and AT16–TiO_2_, respectively. The lower yields at higher TA contents are consistent with increased mass loss from organic-species decomposition during thermal treatment.

### Characterization of Photocatalysts

X-ray diffraction (XRD) patterns were recorded in the 2θ range of 20–80° using a Shimadzu XRD-6000 diffractometer equipped with Cu K_α_ radiation (λ = 1.5418 Å). Measurements were performed at an accelerating voltage of 40 kV and a tube current of 30 mA, with a scanning rate of 1° min^–1^.

N_2_ physisorption isotherms were measured at 77 K using a NOVA 1200e surface area and pore size analyzer (Quantachrome Instruments). The specific surface area (*S*_*BET*_) was determined by the Brunauer–Emmett–Teller (BET) method using adsorption data in the relative pressure range of *P*/*P*_0_ = 0.05 – 0.20:

$$\frac{P}{V\left(P_{0}-P\right)}=\frac{1}{V_{m}C}+\frac{C-1}{V_{m}C}\frac{P}{P_{0}}$$ 1

The monolayer adsorption capacity (V_m_) and BET constant (C) were calculated from the slope (*s*) and intercept (*i*) of the linearized BET plot:

$$V_{m}=\frac{1}{s+i}\;\mathrm{and}\;C=1+\frac{s}{i}$$ 2

The calculated *C* values were approximately 50 for all samples, supporting the suitability of the selected relative-pressure range for BET analysis. The total pore volume (*V*_*t*_) was determined from the N_2_ adsorption volume at *P*/*P*_0_ = 0.99, whereas the micropore volume (*V*_*μ*_) was obtained using the de Boer method.^27^ The mesopore volume (*V*_*meso*_) was calculated as

$$V_{meso}=V_{t}-V_{\mu }$$ 3

The mean pore diameter (*D*_*p*_) was estimated assuming cylindrical pore geometry:

$$D_{P}=\frac{4V_{t}}{S_{BET}}$$ 4

Bright-field transmission electron microscopy (TEM) images were acquired using a JEOL JEM-1400 microscope operated at 120 kV. Scanning electron microscopy (SEM) images were obtained with a FEI Quanta 250 microscope operated at 15 kV and 30 μA. Prior to SEM analysis, the samples were coated with a thin gold layer by sputtering.

Thermogravimetric analysis (TGA) was performed using a Shimadzu TGA-50/50H analyzer. Measurements were conducted from 20 to 800 °C at a heating rate of 10 °C min^–1^ under an argon flow of 50 mL min^–1^ using alumina crucibles.

Diffuse reflectance UV–Vis spectra were recorded using a PerkinElmer Lambda 1050 spectrophotometer. The optical band-gap energies were estimated from Tauc plots^28^ constructed by plotting ${\left[\hbar \upsilon F\left(R\right)\right]}^{1/n}$ *versus* ℏυ where ℏ is Planck’s constant, *υ* is the frequency of the incident radiation, and *n* is related to the nature of the electronic transition. For TiO_2_, *n* = 2 was adopted, corresponding to an indirect allowed electronic transition. The Kubelka–Munk function, (F­(R)), was calculated from the diffuse reflectance data according to eq 5, where (R) is the measured reflectance.^28,29^

$$F\left(R\right)=\frac{{\left(1-R\right)}^{2}}{2R}$$ 5

### Point of Zero Charge (pH_pcz_)

The surface charge properties of the materials were evaluated by dispersing 20.0 mg of adsorbent in 20 mL of 0.1 mol L^–1^ NaCl solution. The initial pH was adjusted over the range of 2–12 using 0.01 or 0.1 mol L^–1^ HCl or NaOH solutions. The suspensions were subsequently stirred at 100 rpm and 25 °C for 24 h to ensure equilibration, followed by filtration and measurement of the final pH of the filtrates.

### Photocatalytic Studies

Photocatalytic activity was evaluated using 6.0 mg of photocatalyst dispersed in 15.0 mL of an aqueous MB solution (10.0 mg L^–1^). Prior to irradiation, the suspension was magnetically stirred in the dark for 30 min to establish adsorption–desorption equilibrium. For each measurement, a 3.0 mL aliquot of the suspension was transferred to a quartz cuvette and subjected to irradiation using a UV-LED system.

The irradiation system comprised a UV-LED (OEM) with a maximum emission wavelength of 365 nm and an output power of 5 mW, positioned above the sample. The suspension was maintained under continuous magnetic stirring, while the temperature was controlled at 25.0 °C using a Peltier device. During irradiation, UV–Vis absorption spectra were acquired over the 400–800 nm range at 1 min intervals, with continuous irradiation maintained throughout the measurements.

## Results and Discussion

### Structural and Thermal Stability Analyses

The crystallographic structure of the synthesized photocatalysts was investigated by X-ray diffraction (XRD), and the corresponding diffraction patterns are presented in Fig. 1. All samples exhibited the characteristic reflections of anatase TiO_2_, demonstrating that the incorporation of TA during synthesis did not induce any phase transformation. The diffraction maxima at 2θ = 25.3°, 37.9°, 47.9°, 54.0°, 55.1°, and 62.8° were indexed to the (101), (004), (200), (105), (211), and (204) crystallographic planes, respectively, in accordance with the standard anatase phase (ICDD PDF No. 21-1272).^30,31^

**Figure 1:**
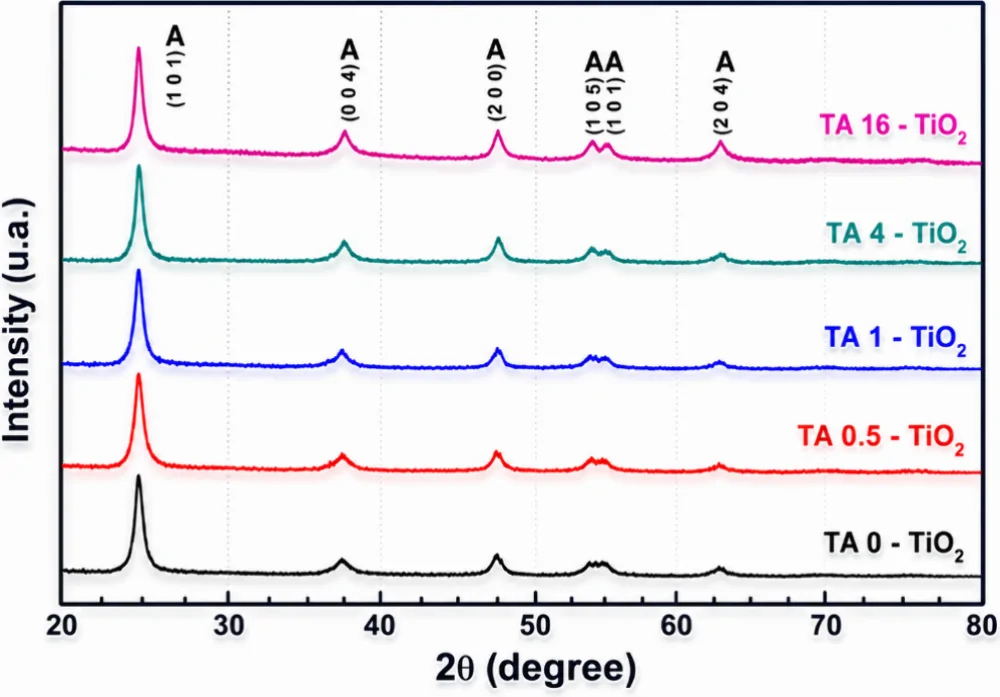
X-ray diffraction (XRD) patterns of TiO_2_ photocatalysts synthesized with varying tannic acid (TA) contents (0.0–16.0), confirming the formation of phase-pure anatase TiO_2_ in all samples.

The absence of additional reflections associated with rutile, brookite, or impurity phases further confirms the formation of phase-pure anatase in all samples. These results demonstrate that the synthesis conditions employed successfully promoted the crystallization of TiO_2_ while preserving the anatase structure regardless of the TA content. To obtain a more detailed understanding of the crystal structure of the prepared TiO_2_, XRD data were fitted to a tetragonal-phase model:^32^

$$\frac{1}{d_{hkl}^{2}}=\frac{\left(h^{2}+k^{2}\right)}{a^{2}}+\frac{l^{2}}{c^{2}}$$ 6

where *a* and *c* are the lattice constants, and *d*_*hkl*_ is the interplanar spacing, which represents the positions of XRD peaks according to Bragg’s law (*λ* = 2*d*_*hkl*_ *sin θ*).

Lattice parameters were determined through linearized least-squares refinement of multiple diffraction peaks using OriginPro 2024. Here, 1/d^2^ was modeled as the dependent variable against the independent variables (h^2^ + k^2^) and l^2^. The refined lattice constants (*a* = 3.786 Å, *c* = 9.484 Å) closely match those of standard anatase TiO_2_, indicating that the crystal structure of the photocatalysts was unaffected by variations in the TA concentration used during synthesis. Furthermore, all samples consistently exhibited a predominant anatase phase. This structural stability aligns with the 450 °C calcination temperature, which falls well below the thermal energy required to induce the anatase-to-rutile transformation (typically >700 °C).^33,34^

The effect of TA content on the composition of the photocatalysts was evaluated by thermogravimetric analysis (TGA), as shown in Fig. 2. The analyses were carried out using untreated samples (TA0, TA0.5, TA1, TA4, and TA16), where the labels correspond to the TA ratio. Three main mass-loss regions were observed, at 120–150 °C, 150–300 °C, and 300–600 °C.

**Figure 2:**
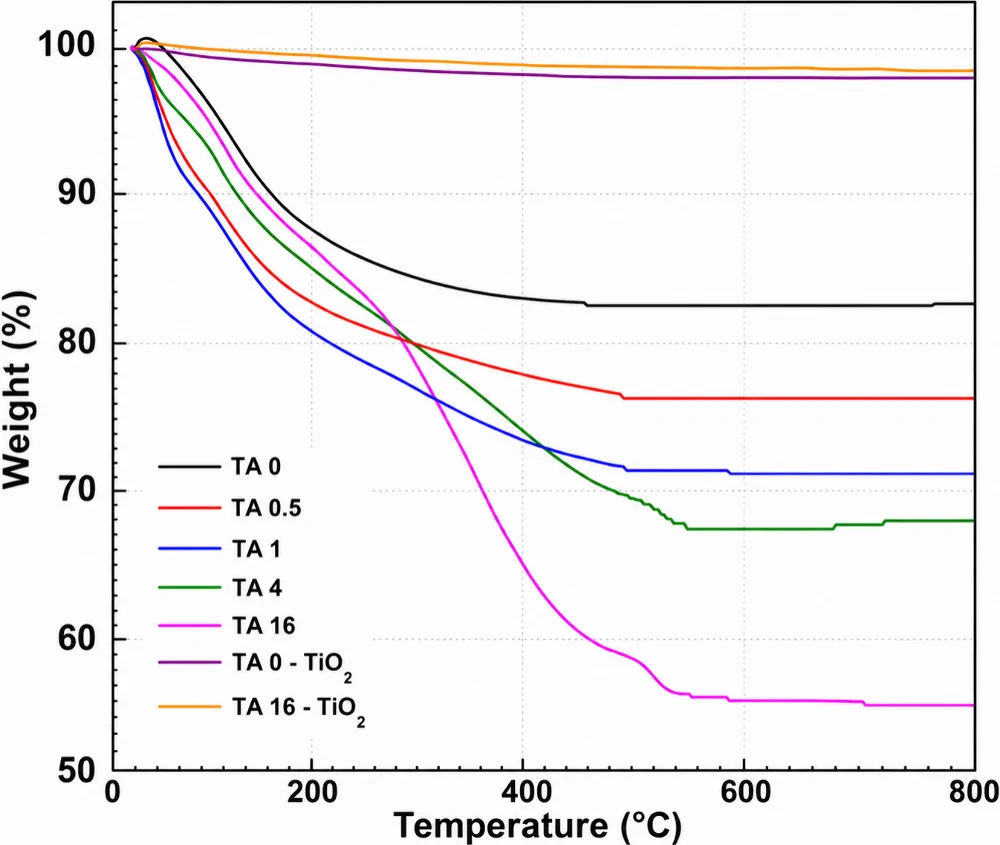
Thermogravimetric analysis (TGA) curves of TiO_2_ samples synthesized with different TA:TTIP ratios, evaluated before and after thermal treatment under an argon atmosphere.

The first region is primarily associated with the removal of physically adsorbed water and residual solvents from the sol–gel synthesis process.^35^ The second region is attributed to the initial thermal decomposition of organic species, mainly TA-derived species and organic–inorganic complexes formed during the synthesis process. The third region is associated with the further decomposition and combustion of the remaining organic fraction. Above this temperature range, the inorganic phase remains comparatively thermally stable, and the residual mass is predominantly associated with TiO_2_.

The total mass loss increased progressively with increasing TA content, from approximately 17% for TA0 to 44% for TA16. The substantially greater mass loss observed for TA16 indicates a higher fraction of thermally decomposable organic species retained in the as-synthesized material at the higher TA ratio. In contrast, the TA0–TiO_2_ and TA16–TiO_2_ reference samples exhibited negligible mass variation over the investigated temperature range, indicating that the pronounced mass losses exhibited by the TA-containing materials arise predominantly from the thermal decomposition of organic species introduced during synthesis. These results demonstrate that TA content strongly influences the organic fraction retained in the as-synthesized materials, which may contribute to the observed changes in textural and optical properties. Accordingly, TA acts not only as a structure-directing agent but also as a major contributor to the organic fraction retained in the resulting materials. The progressive variation in this fraction may influence the structural organization and porosity of the TiO_2_-based materials and, consequently, their photocatalytic behavior.^36,37^

To further evaluate the thermal behavior of the materials, the TGA curves were analyzed through the second derivative d^2^w/dT^2^ allowing identification of the inflection points associated with the maximum decomposition rates. Higher temperatures for the main mass-loss events are generally indicative of greater resistance to thermal degradation.

All samples exhibited a multistep weight-loss profile, indicating the presence of physically adsorbed species and thermally labile organic components. The extent of weight loss increased progressively with TA content, demonstrating that larger amounts of organic matter were incorporated into the precursor network during synthesis. The residual mass at ∼800 °C followed the order TA16 < TA4 < TA1 < TA0.5 < TA0. This trend indicates that both increasing TA content and the incorporation of TiO_2_ significantly influence the thermal behavior of the materials.

TA0.5, TA1, and TA4 exhibited inflection temperatures of 47.9, 54.7, and 53.3°C, respectively, indicating an earlier onset of thermal decomposition. TA0 showed a higher characteristic temperature (∼123.1 °C), indicating improved thermal resistance, whereas TA16 presented the higher value (∼611.3 °C), consistent with a substantially delayed decomposition process and enhanced thermal stability.

The FTIR spectra provide evidence for the interaction of TA with the titanium precursor during the sol–gel process (Fig. 3). A weak band near 3009 cm^–1^ is assigned to C–H stretching vibrations, while the band at approximately 1640 cm^–1^ observed in the synthesized TiO_2_ samples is attributed to the bending vibration of molecularly adsorbed H_2_O and/or surface hydroxyl groups associated with hydrated TiO_2_ surfaces.

**Figure 3:**
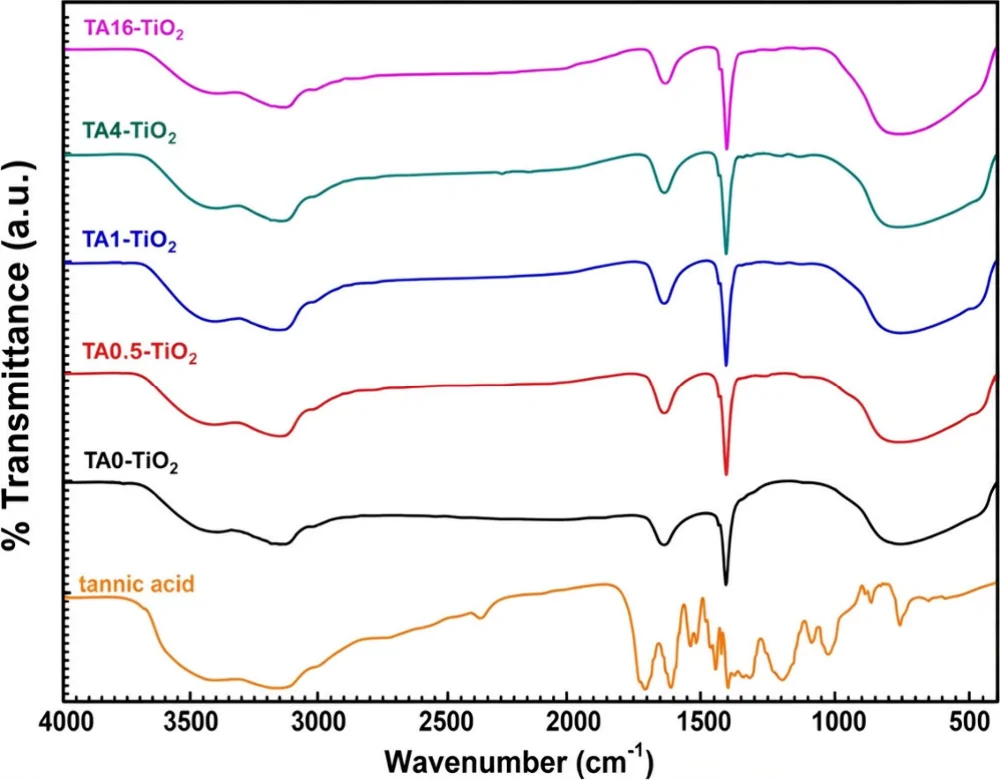
FTIR spectra of TiO_2_ samples synthesized with varying tannic acid contents and calcined under N_2_/O_2_ gas flow, alongside the spectrum of tannic acid used as the precursor.

TA displays characteristic vibrational features near 1200 cm^–1^, associated with C–O stretching of its ester and phenolic functionalities, together with bands in the 1500–1400 cm^–1^ region arising from aromatic C–C and C–O vibrational modes. The aromatic C–H out-of-plane bending bands at approximately 868 and 756 cm^–1^ provide additional spectral signatures of the TA structure. Notably, these characteristic TA bands are substantially attenuated or no longer discernible in the spectra of the synthesized TiO_2_ samples, indicating a pronounced change in the vibrational environment of the TA-derived functional groups during the sol–gel process.

The TiO_2_ samples also exhibit a band near 1400 cm^–1^ that may be associated with Ti–O–C interactions, whereas the broad feature centered at approximately 700 cm^–1^ is characteristic of Ti–O and Ti–O–Ti vibrational modes within the TiO_2_ network. The concurrent attenuation of TA-derived vibrational features and emergence of Ti–O-related bands suggests that oxygen-containing functionalities of TA undergo substantial changes in their chemical environment during precursor hydrolysis and condensation, indicative of their interaction with titanium species rather than simple physical incorporation of the organic component.

These spectral changes are consistent with interactions between TA-derived oxygen functionalities and titanium species during the sol–gel process. Although a definitive distinction between Ti–O–C linkages and closely related Ti–O vibrational environments cannot be made with complete certainty, the observed spectral evolution is consistent with Ti–O–C formation and/or coordination of phenolic oxygen to Ti. The FTIR results therefore provide spectroscopic support for a TA-mediated interaction with the titanium precursor and are consistent with the proposed role of TA as a bio-based structure-directing agent during TiO_2_ formation.^38,39^

The nitrogen adsorption–desorption isotherms of the TiO_2_ samples synthesized with different TA:TTIP ratios are presented in Figure 4. All samples exhibited type IV­(a) isotherms with well-defined hysteresis loops, according to the IUPAC classification, confirming the formation of mesoporous structures. This behavior is characteristic of capillary condensation within pores in the 2–50 nm range and is consistent with the sol–gel synthesis route and the role of TA as a structure-directing agent. In these systems, adsorption is governed by weak, non-directional interactions, comparable to van der Waals forces, between the gas molecules and the TiO_2_ surface, promoting capillary condensation as the relative pressure approaches saturation (*P*/*P*_0_ → 1).

**Figure 4:**
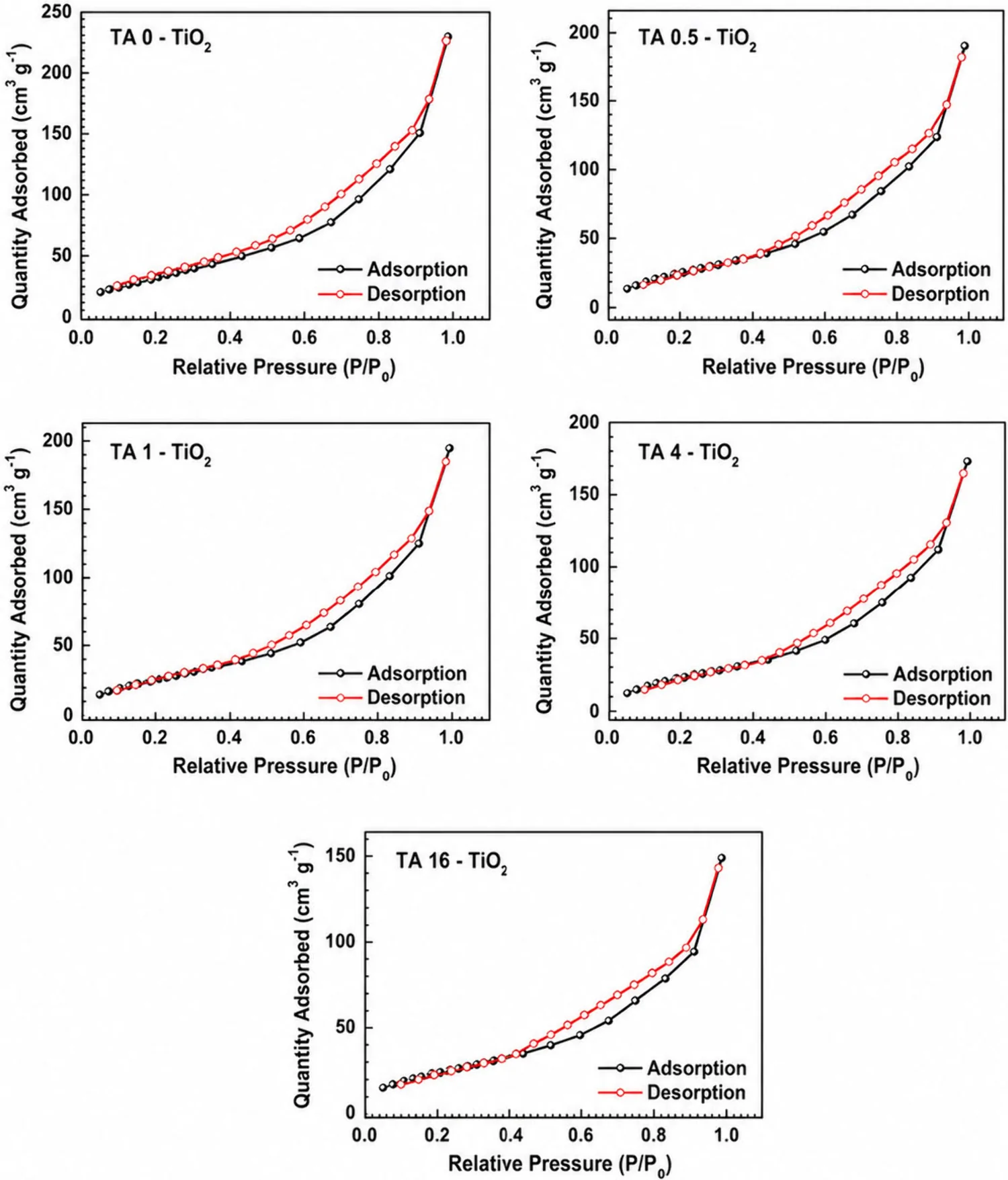
Nitrogen physisorption isotherms (77 K) for the TA-TiO_2_ samples. The hysteretic loops indicate the porous structure evolution as a function of the TA modification level. Adsorption and desorption branches are indicated by the black and red curves, respectively.

At low relative pressures (*P*/*P*_0_ < 0.3), the gradual increase in adsorbed volume is associated with monolayer and subsequent multilayer adsorption on the TiO_2_ surface. In the intermediate relative pressure region (0.4 < *P*/*P*_0_ < 0.9) the pronounced adsorption is attributed to capillary condensation within the mesoporous network. At high relative pressures (*P*/*P*_0_ > 0.9), the sharp increase in adsorption indicates the presence of larger mesopores and interparticle voids formed through aggregation of TiO_2_ nanoparticles.^40,41^

The observed hysteresis loops exhibit H3-type characteristics, indicating the presence of slit-shaped pores associated with TiO_2_ nanoparticle aggregates and interparticle voids. Upon decreasing the relative pressure, desorption occurs along the same path as adsorption, reflecting capillary condensation behavior typical of mesoporous materials. Such features are commonly observed in TiO_2_ synthesized via sol–gel and soft-templating routes, where the removal of organic species generates irregular and interconnected porous networks.

In the present system, TA acts as a transient structure-directing agent, promoting the formation of a hybrid organic–inorganic framework during the sol–gel process. A well-established basis for that statement comes from sol–gel and surfactant/templated synthesis routes of mesoporous TiO_2_, where thermal treatment (calcination) removes organic templates or residual organics, leaving behind porosity and stabilizing the inorganic framework.^41,42^

Fig. S1 presents the linear BET plots obtained from the selected data within the relative pressure range of 0.05–0.20. The corresponding textural properties of the TA:TTIP samples are summarized in Table 2. Nitrogen adsorption–desorption analysis shows that the presence of TA during synthesis significantly influences the textural characteristics of TiO_2_.

**Table 2:** Brunauer–Emmett–Teller (BET) Surface Area and Pore Structure Parameters of TiO_2_ Samples Synthesized with Different TA:TTIP Ratios, Including Specific Surface Area (*S*_BET_), Total Pore Volume (V_t_), Micropore Volume (V_m_), and Average Pore Diameter (D_P_)

Samples	S_*BET*_ (m^2^ g^–1^)	V_t_ (cm^3^ g^–1^)	V_m_ (cm^3^ g^–1^)	D_P_ (nm)
TA0–TiO_2_	123.26	0.35	0.35	11.57
TA0.5–TiO_2_	115.74	0.29	0.29	10.24
TA1–TiO_2_	112.61	0.31	0.31	11.16
TA4–TiO_2_	100.11	0.26	0.26	10.57
TA16–TiO_2_	90.27	0.29	0.29	10.15

In particular, the specific surface area decreases by approximately 27%, from 123 to 90 m^2^ g^–1^, with increasing TA content. This trend suggests that TA modifies the nucleation and growth kinetics of TiO_2_ particles, promoting the formation of larger aggregates and, consequently, reducing the accessible surface area.

In contrast, the average pore diameter remains nearly constant within the 10–11 nm range, indicating that TA acts as a structural modifier, influencing nanoparticle assembly and aggregation during the sol–gel process and subsequent thermal treatment. This behavior is consistent with the predominance of interparticle mesoporosity, which is commonly observed in TiO_2_ materials synthesized via sol–gel routes.^43^ Furthermore, variations in the total pore volume indicate that TA influences the structural organization of the material through the formation of hybrid organic–inorganic networks during synthesis. The subsequent thermal removal of the organic component generates structural voids, contributing to the stabilization of the porous framework. These findings confirm the role of TA as a structure-directing additive that modulates the textural properties of TiO_2_ while preserving its mesoporous character.

The pore size distributions shown in Figure 5 further confirm the predominantly mesoporous nature of the samples, with relatively narrow distributions centered between 3 and 10 nm. This behavior indicates the formation of a homogeneous mesoporous network governed primarily by interparticle voids generated during nanoparticle aggregation in the sol–gel process. Notably, the incorporation of TA preserves the structural integrity of the mesostructure throughout calcination. Furthermore, the position of the pore size distribution maximum remained essentially unchanged among the samples, indicating that TA concentration primarily governs the structural organization of the material.

**Figure 5:**
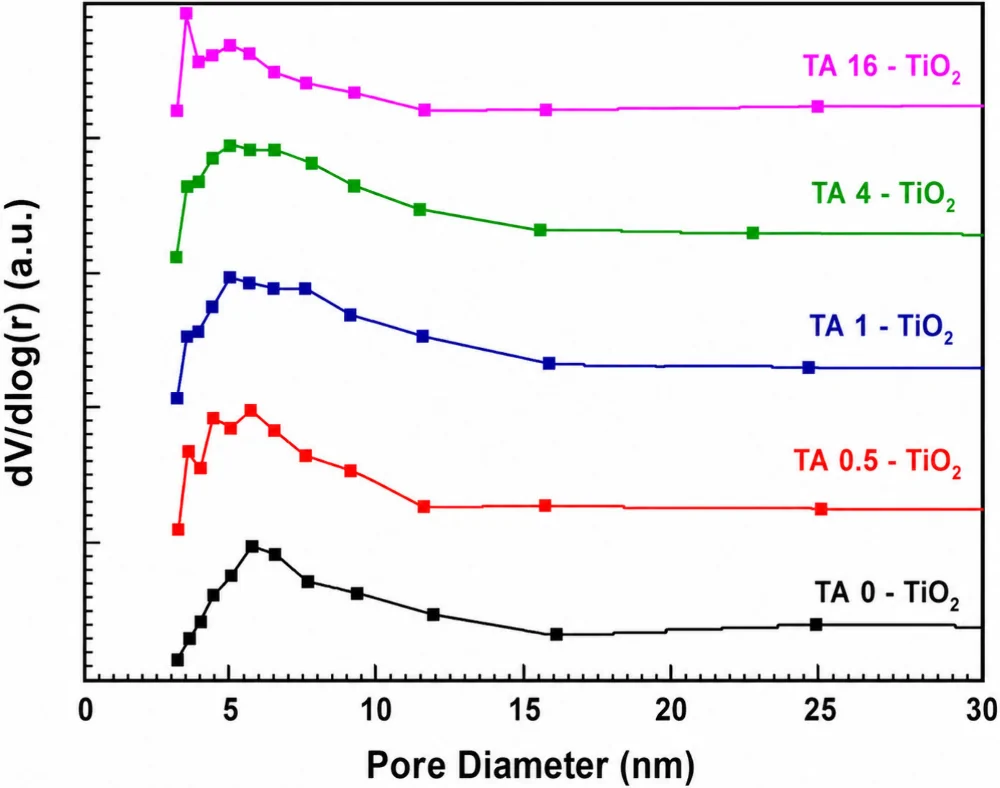
Pore size distribution curves of TiO_2_ samples synthesized with different TA:TTIP ratios, determined by the BJH method from the N_2_ desorption isotherm branch.

Peak pore diameters were determined from the first derivative of the pore size distribution curves $\left(\frac{d}{\mathrm{dD}}\left(\frac{\mathrm{dV}}{d\log\left(r\right)}\right)=0\right)$ of the pore size distribution curves. The resulting peak diameters for TA0, TA0.5, TA1, TA4, and TA16 were 6.3, 6.2, 6.4, 6.3, and 6.1 nm, respectively, indicating that the TA concentration has a negligible effect on the primary pore size.

### Morphology Properties

TEM images of the TiO_2_ samples are presented in Fig. 6. All samples consist of nearly spherical nanoparticles with relatively homogeneous size distribution and a pronounced tendency toward aggregation, a characteristic feature of sol–gel-derived materials.

**Figure 6:**
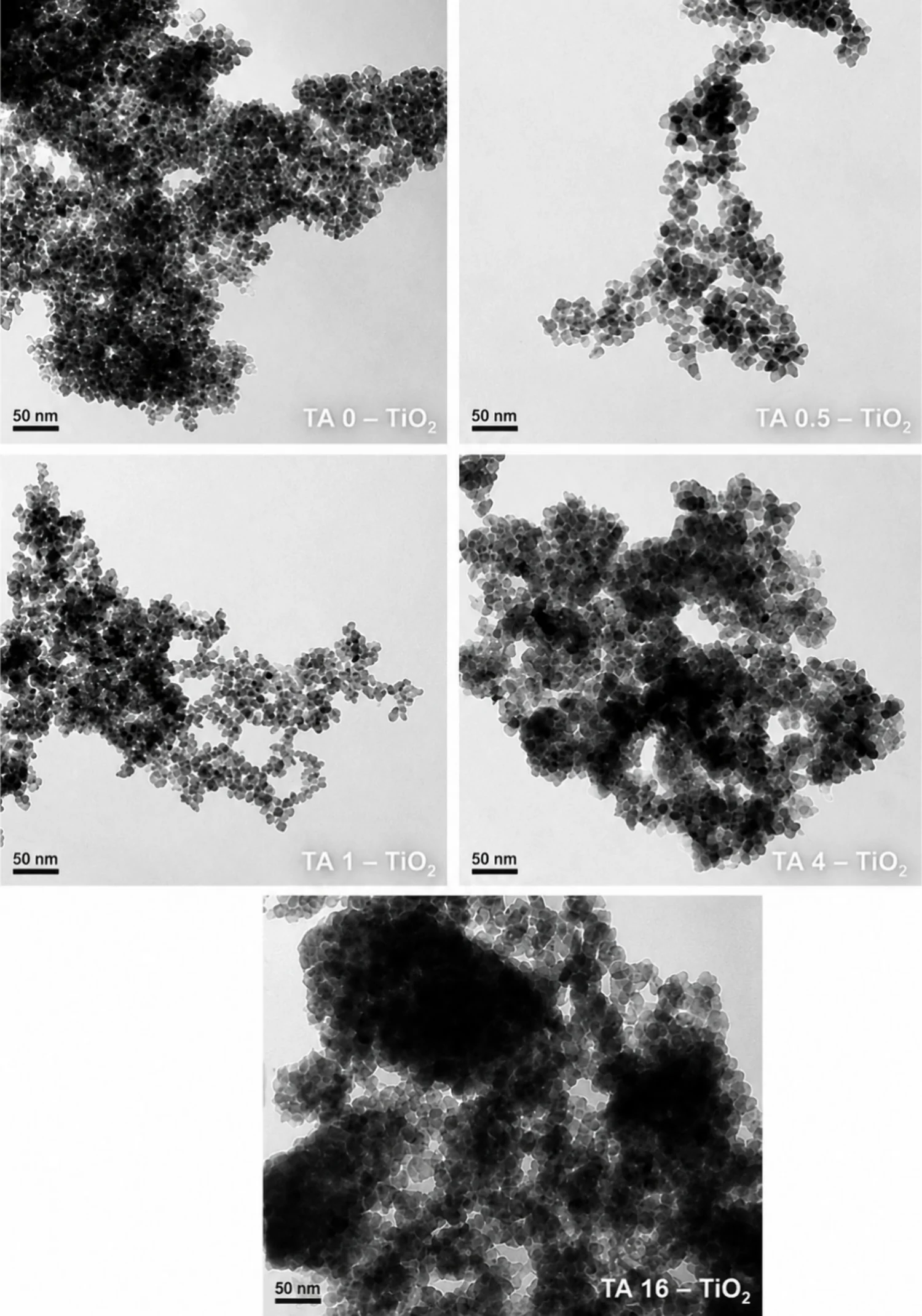
Transmission electron microscopy (TEM) micrographs of TiO_2_ samples prepared using different tannic acid-to-titanium isopropoxide ratios. All images are shown at the same magnification (scale bar = 50 nm).

During synthesis and subsequent thermal treatment, the primary nanoparticles undergo assembly into aggregated structures, giving rise to interparticle mesoporosity. This structural organization corroborates the textural results and further demonstrates that TA influences the nucleation, growth, and assembly of TiO_2_ nanoparticles.^44^

SEM images of the TiO_2_ samples are presented in Figure 7. The TA0–TiO_2_ sample synthesized in the absence of TA (Figure 7a) exhibits irregular agglomerates composed of densely packed nanoparticles with heterogeneous surface morphology, characteristic of sol–gel-derived TiO_2_ materials. Such structures arise from the spontaneous aggregation of primary particles during drying and calcination, leading to secondary aggregates with limited structural ordering.

**Figure 7:**
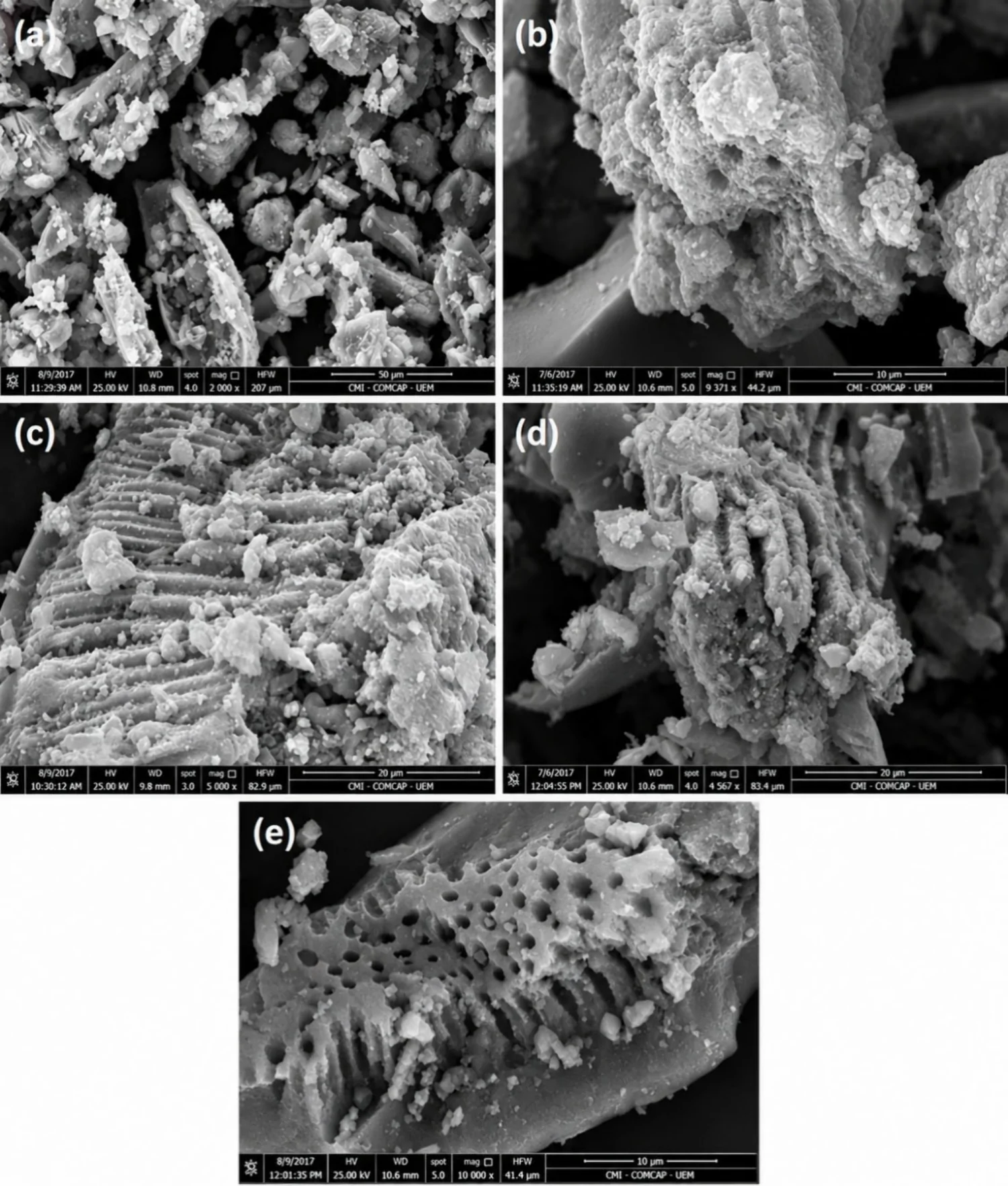
Scanning electron microscopy (SEM) micrographs of TiO_2_ samples synthesized with different TA:TTIP ratios: (a) TA0–TiO_2_, (b) TA0.5–TiO_2_, (c) TA1–TiO_2_, (d) TA4–TiO_2_, and (e) TA16–TiO_2_.

For the samples synthesized in the presence of TA (Figure 7b–e), the morphology remains dominated by interconnected aggregates, although noticeable differences in particle organization and packing density are observed. The presence of TA influences the hydrolysis and condensation of the titanium precursor through interactions between phenolic groups and inorganic species, promoting the formation of a transient organic–inorganic network during synthesis. Following thermal treatment, removal of the organic component generates interconnected aggregates with enhanced structural organization and interparticle voids.

These observations indicate that TA does not alter the aggregation-driven formation mechanism of TiO_2_, but rather modulates the arrangement and packing of the resulting aggregates. The interparticle porosity observed is consistent with the mesoporous structure inferred from the N_2_ adsorption–desorption analyses.

### Photocatalytic Analyses

The coupling of a large number of atomic orbitals in a solid generates *quasi*-continuous energy bands separated by forbidden energy regions, known as band-gaps (E_g_). The photocatalytic mechanism involves the simultaneous oxidation of adsorbed water molecules by photogenerated holes (h^+^) and the reduction of dissolved oxygen by photogenerated electrons (e^–^).^45^ In semiconductors, the width of the energy bands is directly related to the strength of orbital interactions between neighboring atoms. Consequently, the presence of a large forbidden energy region hinders electron excitation from the valence (E_VB_) band to the conduction band (E_CB_), limiting charge carrier generation under low-energy irradiation.^46,47^

In intrinsic semiconductors, the energy difference between the valence band and the conduction band is sufficiently small that thermal excitation can promote electrons across the band-gap, generating electron–hole pairs.^48^ However, TiO_2_ possesses a relatively wide band-gap,^49^ and the thermal energy available at room temperature is insufficient to excite a significant number of electrons from E_VB_ to E_CB_.^48^ Consequently, under ambient conditions and in the absence of illumination, pristine TiO_2_ behaves essentially as an electrical insulator, exhibiting a negligible concentration of thermally generated charge carriers.

Therefore, the primary mechanism for generation of holes in TiO_2_ is photon-induced excitation, typically under ultraviolet (UV) irradiation. This process may be further intensified through templating-agent-mediated approaches that effectively narrow the band-gap energy. Simultaneously, structure-directing agents can engineer defect-rich architectures and intermediate electronic states that facilitate charge transport via lower-energy pathways, thereby promoting charge-carrier mobility and enhancing photocatalytic efficiency.

Upon UV irradiation with photon energy (E_photon_) equal to or greater than the band-gap energy E_g_ of TiO_2_, electrons are promoted from E_VB_ to E_CB_, generating electron–hole (e^–^/h^+^) pairs. For pure TiO_2_, this excitation typically occurs for wavelengths (λ_g_) shorter than approximately 387 nm, corresponding to the ultraviolet region, according to (TiO_2_ + hν → e^–^ + h^+^).^50^

In TA-directed TiO_2_, the organic additive does not behave as a conventional substitutional dopant; rather, it may modulate the structural and electronic landscape by promoting defect formation and influencing mesostructural evolution. Thermal treatment promotes the formation of Ti–O–C^51^ and C–O^52^ surface species, oxygen vacancies, and localized electronic states within the band structure.^53,54^ These defect-induced intermediate levels enable electronic transitions through sub-band-gap states (E_VB_ → defect states → E_CB_), thereby reducing the excitation energy required compared with pure TiO_2_.

As a result, the TA-mediated material may exhibit an apparent narrowing of the effective band-gap energy due to the formation of defect-related electronic states, extending light absorption toward the visible region. In addition, the retained TA-derived organic fraction may contribute to interfacial charge-transfer processes and thereby influence the electronic properties of the TiO_2_-based materials. Under irradiation, the organic component may also act as a photosensitizer, promoting electron transfer to the conduction band of TiO_2_ (TA + hv → TA*), followed by electron injection into TiO_2_ (TA* → TA^+•^ + e^–^).^55^

Carbon species derived from TA may act as electron-trapping sites, enhancing spatial separation of photogenerated e^–^/h^+^ pairs, suppressing recombination, and extending carrier lifetimes, thereby facilitating charge migration to the catalyst surface. The photogenerated electrons subsequently participate in the reduction of adsorbed oxygen to superoxide radicals (O_2_ + e^–^ → O_2_^•–^), whereas the corresponding holes drive the oxidation of water or hydroxide ions, producing highly reactive hydroxyl radicals (H_2_O + h^+^ → HO^•^ + H^+^; HO^–^ + h^+^ → HO^•^). These reactive oxygen species play a central role in the oxidative degradation of organic pollutants.^4,56,57^

The band-gap energy values were estimated from the diffuse reflectance spectra by Tauc plot extrapolation (Fig. 8). The corresponding absorption edge wavelengths (λ_g_ = hc/E_g_, where h = 6,626 × 10^–34^ Js, and c = 2,998 × 10^8^ ms^–1^) are summarized in Table 3. The UV-LED emission (≈3.40 eV) provides sufficient photon energy to excite P25 and all synthesized TiO_2_ samples, whose band-gap energies are lower than the incident photon energy.

**Figure 8:**
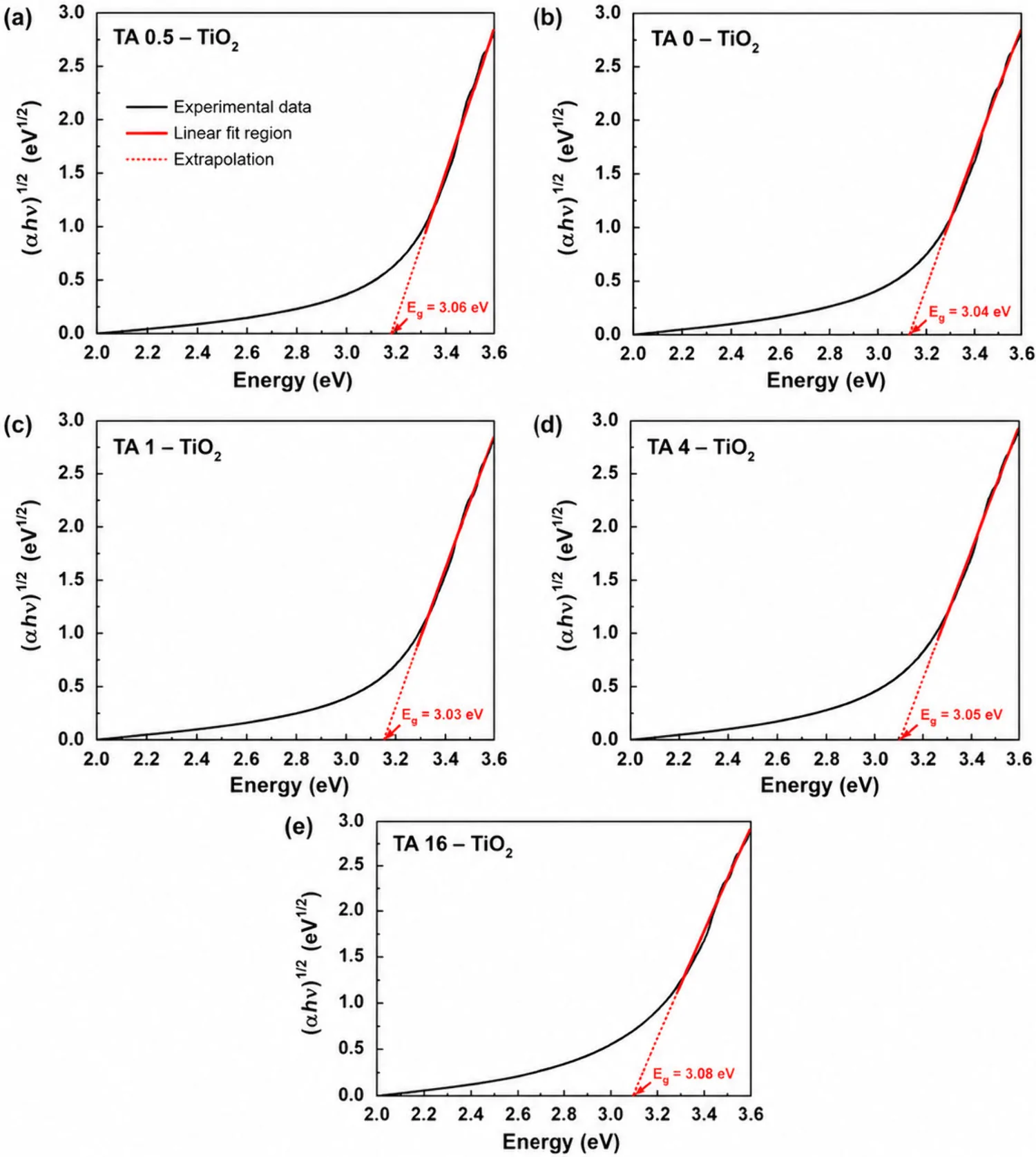
Tauc plots obtained from diffuse reflectance spectroscopy (DRS) measurements of TiO_2_ samples synthesized at different TA:TTIP ratios: (a) TA0.5–TiO_2_, (b) TA0–TiO_2_, (c) TA1–TiO_2_, (d) TA4–TiO_2_, and (e) TA16–TiO_2_, used to estimate the optical band-gap energies.

**Table 3:** Optical Band Gap Energies (E_g_), Corresponding Absorption Wavelengths (λ_g_), and Conduction- and Valence-Band Edge Potentials (E_VB_ and E_CB_, Respectively) of TiO_2_ Samples Synthesized with Different TA:TTIP Molar Ratios^a^

Samples	**E**_**g**_ (eV)	**λ**_**g**_ (nm)	**E**_**CB**_ (V *vs.* NHE)	**E**_**VB**_ (V *vs.* NHE)	**ΔG**_**CB**_^**0**^ (**kJ mol** ^**–1**^)	**ΔG**_**VB**_^**0**^ (**kJ mol** ^**–1**^)
P25 DEGUSSA	3.14	395	–0.26	+2.88	+6.75	–48.24
TA0–TiO_2_	3.04	408	–0.21	+2.83	+11.58	–43.42
TA0.5–TiO_2_	3.06	405	–0.22	+2.84	+10.61	–44.38
TA1–TiO_2_	3.03	409	–0.20	+2.83	+12.06	–42.94
TA4–TiO_2_	3.05	407	–0.21	+2.84	+11.10	–43.90
TA16–TiO_2_	3.08	403	–0.23	+2.85	+9.65	–45.35

a Values were estimated from DRS data using the Kubelka–Munk function and Tauc plots. Band-edge potentials are referenced to NHE.

The progressive decrease in E_g_ and red shift of the absorption edge indicate that the TA-assisted sol–gel process modifies the electronic structure of TiO_2_. Similar band-gap narrowing in carbon-modified and defect-rich TiO_2_ has been associated with additional electronic states that influence light absorption and charge-transfer pathways.^58^ The present results are therefore consistent with TA-induced electronic-state modification, although UV–Vis diffuse reflectance spectroscopy cannot directly resolve specific species such as Ti^3+^ or oxygen vacancies; these features are consequently considered plausible contributors.

To gain deeper insight into the photocatalytic mechanism and evaluate the thermodynamic feasibility of reactive species generation, E_CB_ and E_VB_ edge potentials of the synthesized photocatalysts were estimated and compared with the redox potentials of relevant oxidative and reductive reactions, using the Normal Hydrogen Electrode (NHE) as the reference scale.^45,59^ Such an analysis provides valuable information on whether the photogenerated charge carriers possess sufficient energy to generate reactive oxygen species involved in pollutant degradation.

E_VB_ determines the oxidative capability of photogenerated holes, whereas, governs the reductive capability of photogenerated electrons. Therefore, the relative positions of these bands can be used to predict the capability of the photocatalyst to drive key redox processes under irradiation.

The band-edge potentials were estimated using the absolute electronegativity method, where E_CB_ = χ – E_e_ – E_g_/2 and E_VB_ = E_CB_ + E_g_.^60,61^ Here, χ is the absolute electronegativity of TiO_2_ (5.81 eV), and E_g_ is the free-electron energy on the NHE scale (4.50 eV). The resulting conduction- and valence-band edge potentials are listed in Table 3.

The oxidation potentials of the relevant reactions are approximately HO^–^/HO^•^ = +1.99 and H_2_O/HO^•^ = +2.38 V, whereas the reduction potential of O_2_/O_2_^•–^ is approximately −0.33 V.^4^ As shown in Table 3, all synthesized samples exhibit E_VB_ values ranging from +2.83 to +2.88 V vs NHE, indicating that the photogenerated holes possess sufficient oxidative potential to promote the oxidation of surface HO^–^and/or H_2_O to HO^•^ radicals. Therefore, HO^•^ formation is thermodynamically feasible for all photocatalysts. In addition, direct oxidation of adsorbed MB by photogenerated holes may contribute to the degradation process. Consistently, the ΔG_VB_^0^ values for these oxidation pathways range from −42.94 to −48.24 kJ mol^–1^, supporting their thermodynamic feasibility. Direct oxidation of adsorbed MB by photogenerated holes may also contribute to the degradation process.

In contrast, the estimated E_CB_ values (−0.26 to −0.20 V) are not sufficiently negative to thermodynamically drive the one-electron reduction of O_2_ to O_2_^•–^. Accordingly, the ΔG_CB_^0^ values for O_2_ reduction are positive, ranging from +6.75 to +12.06 kJ mol^–1^, indicating that direct O_2_^•–^ generation through conduction-band electron transfer is thermodynamically unfavorable for the synthesized photocatalysts and is not regarded as a principal pathway in the proposed mechanism. The band-edge analysis is employed here to assess the thermodynamic feasibility of the proposed oxidative pathways rather than to directly identify individual ROS.

Despite the less negative E_CB_, the materials exhibited enhanced activity, which may be associated with the combined effects of their structural, surface, and optical properties. In particular, the red-shifted absorption edge may improve visible-light harvesting, while the increased specific surface area and greater availability of surface-active sites^62^ may enhance MB adsorption and facilitate interfacial oxidation reactions.

Photocatalytic activity likely reflects the interplay of visible-light utilization, charge-carrier dynamics, surface properties, adsorption, and interfacial oxidative processes, although the specific contribution of charge-carrier separation and recombination cannot be established without direct measurements. The proposed oxidation pathways involving HO^•^ and direct hole transfer are therefore considered thermodynamically feasible rather than experimentally demonstrated.

The thermodynamic driving forces associated with the generation of reactive oxygen species were evaluated from the Gibbs free energy change according to

$$\Delta G^{{}^{\circ}}=-nF\Delta E^{o}$$ 7

where *n* is the number of electrons involved in the redox process, *F* is the Faraday constant (96485 *C mol* ^–1^), and Δ*E* ^*o*^ is the potential difference between the semiconductor band-edge potential and the corresponding redox potential. Based on this relationship, the Gibbs free energy changes associated with the reduction of dissolved oxygen to superoxide radicals (O_2_^•–^) and the oxidation of water to hydroxyl radicals (HO^•^) were calculated as follows:^63^

$$\Delta G_{\mathrm{CB}}^{0}=-\mathrm{nF}\left(E_{0_{2/O_{2}^{•-}}}^{0}-E_{\mathrm{CB}}^{o}\right)$$ 8

$$\Delta G_{\mathrm{VB}}^{0}=-\mathrm{nF}\left(E_{\mathrm{VB}}^{0}-E_{H_{2}O/HO^{•}}^{o}\right)$$ 9

where E_O_2_/O_2_^•–^_^o^ = −033 V and E_H_2_O/HO^•^_^o^ = +2.38 V *versus* NHE. Negative Δ*G* ^0^ values indicate thermodynamically favorable charge-transfer processes, whereas positive values denote thermodynamically unfavorable reactions. Consequently, the magnitude and sign of ΔG_VB_^0^ provide a quantitative measure of the thermodynamic feasibility of superoxide and hydroxyl radical generation, respectively (Table 3).

The positive ΔG_CB_^0^ values indicate that the reduction of O_2_ to O_2_^•–^ is thermodynamically unfavorable for all samples, becoming progressively less favorable with increasing TA content. This behavior is attributed to the positive shift of the conduction band edge, which reduces the reducing power of photogenerated electrons. Conversely, the negative ΔG_VB_^0^ values confirm that the oxidation of H_2_O to HO^•^ remains thermodynamically favorable. However, the decreasing magnitude of ΔG_VB_^0^ reveals a gradual loss of oxidative driving force due to the downward shift of the valence band edge. Therefore, although TA incorporation narrows the band-gap and extends visible-light absorption, it simultaneously reduces the intrinsic redox capability of TiO_2_, particularly for superoxide and hydroxyl radical generation.

### Degradation Studies

ROS may contribute to photocatalytic degradation, particularly highly reactive species such as HO^•^. Based on the band-edge analysis, the proposed mechanism primarily involves photogenerated-hole-mediated surface oxidation and subsequent HO^•^ formation, whereas O_2_^•–^ generation is not considered an established pathway. Thus, the photocatalytic activity likely reflects the interactions among light harvesting, charge-carrier dynamics, surface properties, and interfacial oxidation processes.

Due to their high oxidation potential (E^•^ ≈ +2.8),^57^ the HO^•^ radical preferentially attacks electron-rich domains of MB via electrophilic addition and electron-transfer mechanisms. As depicted in Fig. 9, the conjugated C–S and C–N linkages within the phenothiazine chromophore constitute the primary sites for oxidative attack, leading to disruption of the chromophoric structure.

**Figure 9:**
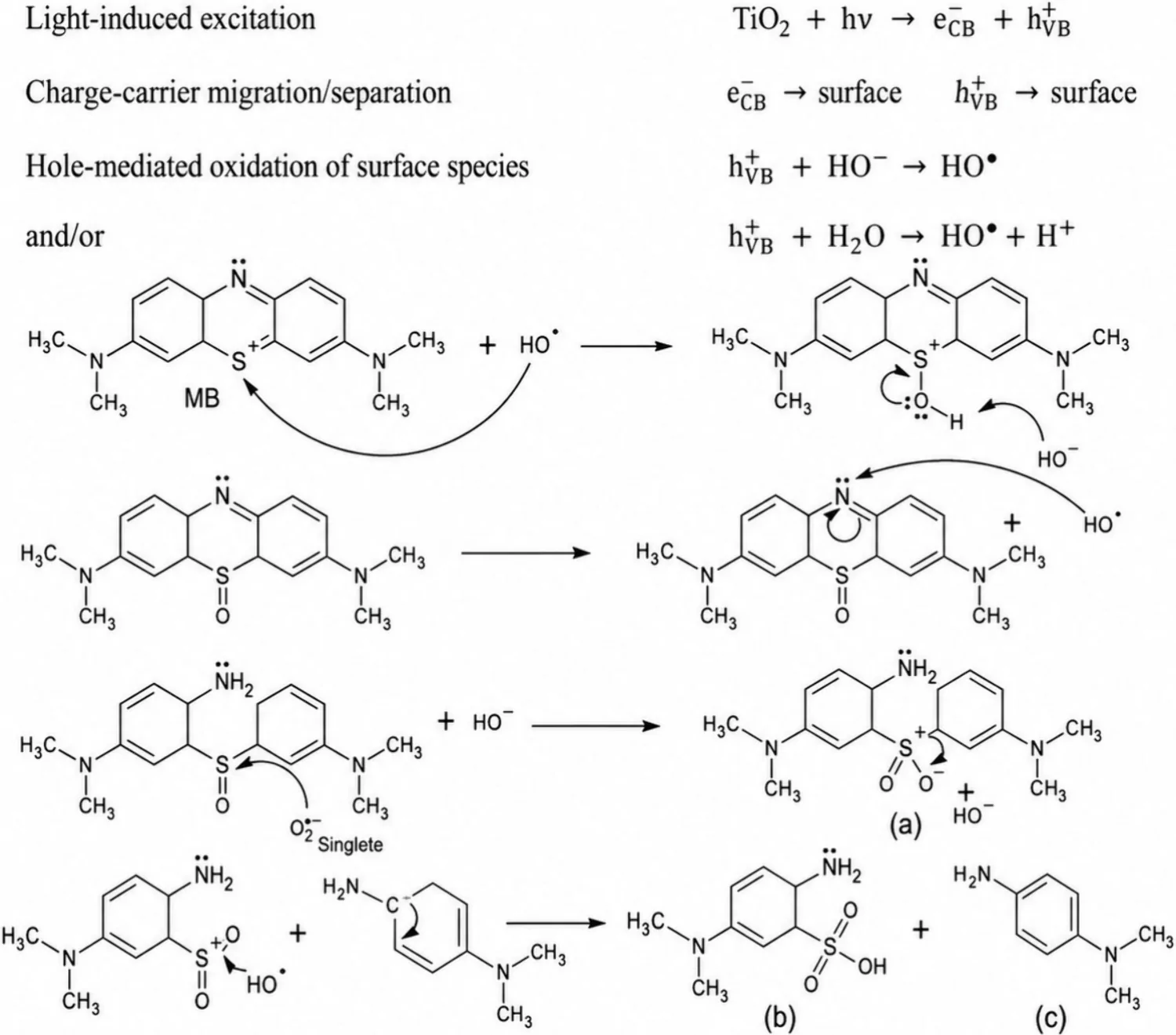
Proposed photocatalytic degradation pathway based on band-edge analysis, showing HO^•^ generation and hole-mediated oxidation of methylene blue. Direct reduction of O_2_ to O_2_^•–^ is excluded based on the calculated E_CB_ values. Oxidation at the sulfur center may alter the conjugated π-electron system, promoting C–S bond cleavage and ring opening to form smaller, colorless intermediates: (a) 2-{[3-(dimethyl­amino)­cyclo­hexa-2,4-dien-1-yl]­sulfinyl}-*N4,N4*-dimethyl­benzene-1,4-diamine, (b) 2-amino-5-dimethyl­amino­benzene­sulfonic acid anion, and (c) dimethyl-4-phenylene­diamine.

Although the aromatic ring participates in a delocalized π-system, the sulfur and nitrogen heteroatoms exert a strong electronic influence through their lone-pair electrons, thereby redistributing charge density within the conjugated framework. Consequently, oxidation is initiated preferentially at these heteroatom-associated sites, leading to destabilization of the chromophoric structure, bond scission, and progressive degradation of the dye molecule.

The formation of the sulfone group (−SO_2_) strongly withdraws electron density from the adjacent aromatic carbons, rendering them highly electrophilic. HO^•^ radicals subsequently attack these electron-deficient carbon centers, promoting cleavage of the carbon–sulfur (C–S) bond and thereby inducing opening of the central heterocyclic ring.

Additional reactive species and dissolved oxygen may also contribute to the degradation of the sulfur-containing moiety and the conjugated π-electron system (chromophore) associated with the visible-light absorption at 663 nm. This oxidative transformation of the conjugated structure yields smaller, colorless aromatic intermediates, including (a) 2-{[3-(dimethyl­amino)­cyclohexa-2,4-dien-1-yl]­sulfinyl}-*N*4,*N*4-dimethylbenzene-1,4-diamine, (b) 2-amino-5-dimethyl­amino­benzenesulfonate, and (c) N,N-dimethyl-1,4-phenylenediamine.^45^

The surface acid–base properties of the TA–TiO_2_ photocatalysts can influence MB adsorption and subsequent interfacial photocatalytic reactions. The determined pH_pcz_ values ranged from 6.18 to 6.50 (Table S1), indicating that the photocatalyst surfaces are predominantly positively charged at pH < pH_pcz_ and negatively charged at pH > pH_pcz_. Thus, under alkaline conditions, electrostatic attraction between the negatively charged surface and cationic MB can enhance dye adsorption and its interfacial concentration, potentially favoring photocatalytic degradation. In contrast, at pH values below the pH_pcz_, surface protonation is expected to decrease the electrostatic affinity for MB. Therefore, surface charge governed by protonation–deprotonation equilibria may influence MB adsorption and photocatalytic activity, although other pH-dependent factors, including changes in surface speciation and charge-transfer processes, may also contribute.

### UV-LED-Induced Photocatalytic Degradation

The UV-LED emission maximum at 368 nm overlaps with the excitation region of the synthesized TiO_2_ materials, as shown in Figure 10. Because MB exhibits its principal absorption band near 664 nm, direct dye excitation under these conditions is expected to be limited, favoring excitation of the semiconductor. TA0–TiO_2_, TA1–TiO_2_, and TA4–TiO_2_ exhibited comparable spectral responses, with higher intensities and narrower overlap with the emission profile than TA0.5–TiO_2_ and TA16–TiO_2_. Increasing TA content was accompanied by decreased spectral intensity and band-gap energy, suggesting that the organic precursor modulates the electronic and optical properties of TiO_2_.

**Figure 10:**
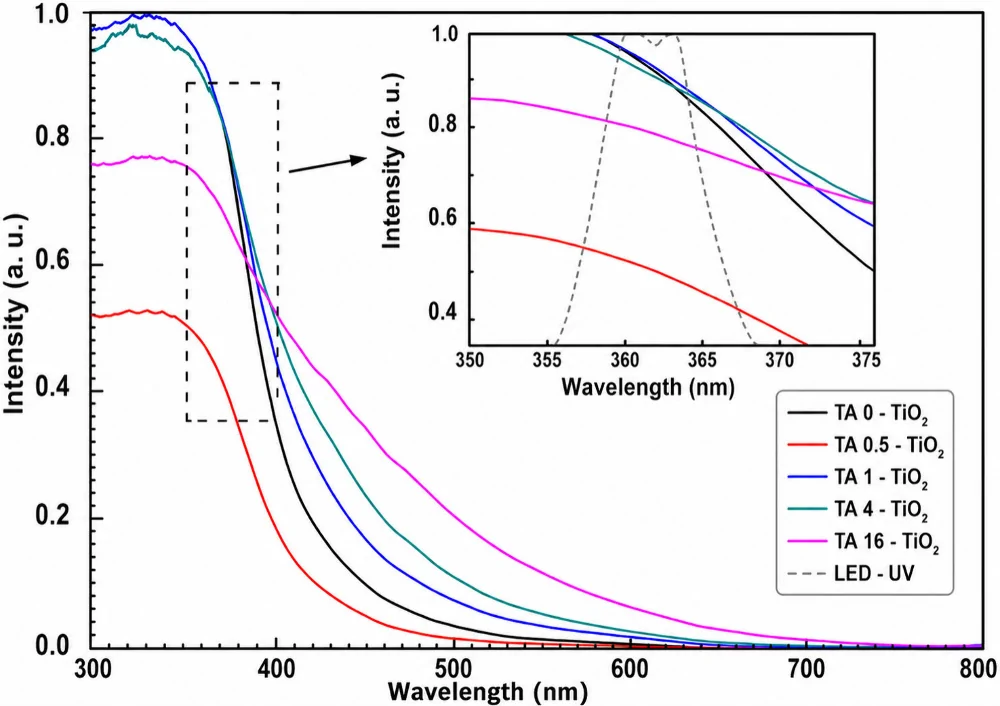
Spectral overlap between the UV-LED emission profile and the UV–vis absorption spectra of the synthesized materials, highlighting the potential excitation of the TiO_2_-based photocatalysts under the irradiation conditions employed.

A dark equilibration step was performed before irradiation, with each suspension stirred for 30 min to establish adsorption–desorption equilibrium. Irradiation was then initiated, and the MB concentration was monitored spectrophotometrically at 1 min intervals for 230 min. Accordingly, the kinetic profiles in Fig. 11 represent the irradiation period only. The photolysis control exhibited only a slight decrease in MB concentration, yielding an apparent rate constant of 0.0005 min^–1^, substantially lower than those of TA0–TiO_2_ (0.0160 min^–1^) and TA0.5–TiO_2_ (0.0203 min^–1^). These results indicate a substantial contribution of TiO_2_-assisted photoinduced processes to MB removal under irradiation, while direct photolysis plays a minor role.

**Figure 11:**
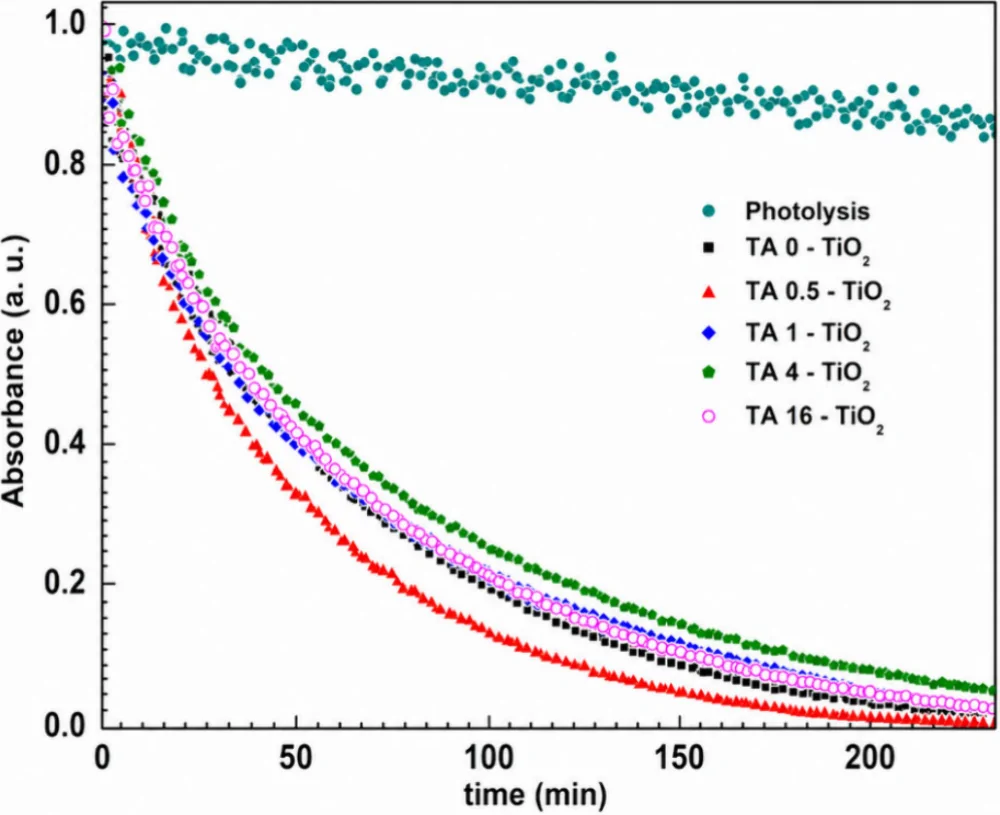
Photocatalytic degradation profiles of methylene blue under UV-LED irradiation using TiO_2_ photocatalysts synthesized with different TA:TTIP molar ratios.

TA0–TiO_2_ exhibited an absorption edge extending into the visible region despite the absence of TA, suggesting that intrinsic defect states generated during the sol–gel process may contribute to its optical response. Increasing TA content progressively red-shifted the absorption edge; however, this extension of visible-light absorption did not translate directly into enhanced MB removal, indicating that optical absorption alone does not determine the observed activity.

TA0.5–TiO_2_ showed the highest apparent removal rate. This behavior cannot be attributed solely to surface area, which decreased with increasing TA content, but may instead arise from the combined effects of structural features, surface properties, defect states, and reactive-site accessibility induced by TA-assisted synthesis. Beyond this composition, further TA incorporation did not improve the removal rate, suggesting that additional structural and surface modifications do not necessarily translate into greater interfacial reactivity.

As MB is a photosensitive dye, a contribution from dye sensitization cannot be excluded. The results are therefore interpreted as evidence of an enhanced photocatalytic contribution to irradiation-assisted MB removal rather than as definitive evidence for a single degradation pathway.

Similar behavior has been reported for hybrid photocatalytic systems, in which excessive incorporation of secondary components can induce light-shielding effects and hinder access to reactive sites, thereby limiting photocatalytic performance.^58^ The results indicate that an intermediate TA content provides a favorable combination of optical and interfacial characteristics for MB removal. Rather than maximizing a single material property, the photocatalytic response appears to depend on the combined effects of these factors, underscoring the importance of controlling TA incorporation during synthesis.

### Kinetic Studies

The kinetic parameters for MB photodegradation under UV-LED irradiation are summarized in Table 4. All experiments were conducted in triplicate, and the strong linear correlation obtained from the pseudo-first-order model supports first-order kinetics under the investigated conditions:

$$v_{0}=k\left[MB\right]\left[{HO}^{•}\right]$$ 10

where v_0_ and k represent the initial degradation rate and intrinsic rate constant, respectively,

**Table 4:** First-Order Kinetic Parameters for Methylene Blue Photodegradation under UV-LED Irradiation

*Photocatalysts*	*k* (*min* ^–1^)	*t*_1/2_ (*min*)	*v*_0_ (*mg L* ^–1^ *min* ^–1^)	*R* ^2^	SE of k (*×*10^–5^ *min* ^–1^)
MB Photolysis	0.0005	1386.29	0.005	0.896	1.12
TA0–TiO_2_	0.0160	43.10	0.160	0.996	6.44
TA0.5–TiO_2_	0.0203	34.07	0.203	0.994	9.86
TA1–TiO_2_	0.0125	55.72	0.125	0.997	3.89
TA4–TiO_2_	0.0124	55.89	0.124	0.997	3.80
TA16–TiO_2_	0.0147	46.99	0.147	0.998	3.74

Oxidation of surface-bound H_2_O or HO^–^ species by photogenerated holes can contribute to the formation of HO^•^ radicals, which participate in the degradation of MB. Because these reactive species are continuously generated under irradiation, their effective concentration can be considered approximately constant relative to that of MB over the reaction time scale and therefore incorporated into the apparent rate constant (*k*′). Thus, *k*′ reflects the combined contribution of the coupled photocatalytic processes rather than a single elementary reaction step, as described by eq 11.

$$k^{\prime }=k\left[{HO}^{•}\right]$$ 11

Substitution of eq 11 into eq 10 gives

$$v_{0}=k^{\prime }{\left[MB\right]}_{0}$$ 12

where *v*_0_ corresponds to the initial degradation rate in the TiO_2_-mediated photocatalytic process.

In the reference experiment, photolysis exhibited a prolonged half-life (*t*_1/2_ = 1386.29 min), indicating a limited contribution of direct photolysis to MB degradation. In contrast, all synthesized TiO_2_ samples displayed substantially higher degradation rates, highlighting their pronounced photocatalytic activity toward MB removal.

TA0.5–TiO_2_ exhibited the highest photocatalytic activity, with a half-life of 34.07 min. This behavior suggests that a low TA content induces subtle structural and textural modifications that favor MB adsorption and interfacial reactions. The enhanced performance may arise from the combined effects of structural organization, accessible surface sites, and interparticle porosity, which could facilitate the generation and transport of reactive oxygen species under UV irradiation. Comparable trends have been reported for engineered TiO_2_-based photocatalysts, in which increased surface area and improved adsorption promote interfacial charge-transfer processes and faster degradation kinetics.^64^

The *v*_0_ values were determined assuming an initial MB concentration of 10 mg L^–1^. TA0.5–TiO_2_ displayed an initial degradation rate of 0.203 mg L^–1^ min^–1^, representing a 27% enhancement relative to pristine TiO_2_ and an approximately 41-fold higher rate than that observed under photolysis alone. These results demonstrate that the effectiveness of TA is strongly dependent on its concentration during synthesis, revealing a distinct structure–property–activity relationship. The enhanced photocatalytic performance of TA0.5–TiO_2_ can be attributed to the favorable interplay among its textural characteristics, surface accessibility, light absorption, and charge-carrier dynamics. The resulting acceleration in degradation kinetics suggests more effective use of the incident radiation.^65,66^

Importantly, TA16–TiO_2_ exhibited a narrower band gap than TA0.5–TiO_2_ but showed lower photocatalytic activity, demonstrating that enhanced light absorption alone is insufficient to maximize photocatalytic performance. At excessive TA concentrations, structural changes may promote aggregation and densification of the TiO_2_ network, reducing surface accessibility and impairing charge-transfer processes. The results therefore indicate that photocatalytic performance depends on the interplay among light harvesting, charge-carrier dynamics, surface accessibility, and structural organization, rather than on optical properties alone.

To place these findings in context, Table 5 compares the photocatalytic performance of the synthesized materials with representative TiO_2_-based systems reported in the literature. A wide range of strategies has been explored to improve TiO_2_ photocatalysis, including heterostructure engineering, incorporation of TA-derived organic fractions, and surface or textural modification. For example, F–TiO_2_/LDO heterostructures improve charge separation and reduce the band-gap energy, although their photocatalytic rates remain relatively limited. TiO_2_/RGO composites, in contrast, facilitate electron transport and increase surface area, leading to improved photocatalytic activity. Polymer-modified systems such as PPy–TiO_2_ can likewise narrow the band gap; however, excessive polymer loading may induce light-shielding effects that limit photon utilization.^58^ Other approaches, such as silica-supported TiO_2_, rely primarily on increased surface area and adsorption capacity to enhance photocatalytic performance.^64^

**Table 5:** Comparison of Synthesis Strategies, Physicochemical Characteristics, and Photocatalytic Performance of TiO_2_-Based Photocatalysts for Methylene Blue Degradation, Including Materials Reported in the Literature and Those Developed in the Present Work

Photocatalyst	Method	Template Modifier	SBET (m^2^ g^–1^)	Band gap (eV)	Light source	*k* (min^–1^)	Ref.
TiO_2_	Sol–gel	None	–	3.30	UV	0.0145	^67^
TiO_2_ (P25)	Commercial	None	–	3.30	UV	0.0046	^67^
TiO_2_ (P25 dispersed)	Ultrasonic dispersion	None	–	–	UV (365 nm)	–	^68^
F-TiO_2_/LDO	Hydrothermal + calcination	F doping + ZnAl-LDO heterojunction	70.83	2.78	Xe lamp (>320 nm)	0.00412	^65^
TiO_2_/mRH-SNP	Sol–gel + magnetization	SiO_2_ (rice husk) + Fe_3_O_4_	144.06	3.20	LED	–	^64^
PPy–TiO_2_	Mechanical mixing	Polypyrrole	–	2.50	UV/vis	–	^58^
TiO_2_-coated membrane	Sol–gel + dip-coating	Glass membrane support	–	-	UV	–	^69^
TiO_2_/RGO (5%)	Hydrothermal (in situ)	Reduced graphene oxide	96.6	3.05	UV/sunlight	0.031	^70^
TA0.5–TiO_2_	Sol–gel	Tannic acid	115.74	3.06	UV-LED	0.0203	This work
TA1–TiO_2_	Sol–gel	Tannic acid	112.61	2.99	UV-LED	0.0124	This work
TA4–TiO_2_	Sol–gel	Tannic acid	100.11	2.97	UV-LED	0.0124	This work
TA16–TiO_2_	Sol–gel	Tannic acid	90.27	2.79	UV-LED	0.0147	This work

Unlike approaches that rely primarily on heterojunction formation, guest-species incorporation, or organic–inorganic hybridization, the present strategy employs TA as a bio-based molecular structure-directing agent to modulate the TiO_2_ framework during sol–gel formation. The resulting photocatalytic behavior demonstrates that controlled structural and textural organization can be achieved without deliberately incorporating additional elements into the TiO_2_ lattice, while still substantially modulating the photocatalytic properties of the material. Therefore, the significance of the present approach lies not simply in the use of TA as a sustainable additive, but in its ability to establish a concentration-dependent structure–property–activity relationship that can be exploited for the rational design of mesoporous TiO_2_ photocatalysts.

## Conclusions

In summary, TA-assisted sol–gel synthesis provides a sustainable route for tailoring the structural, textural, and electronic characteristics of TiO_2_ photocatalysts. TA promoted the formation of mesoporous anatase and modulated its surface and optical properties in a concentration-dependent manner. The enhanced photocatalytic performance resulted from the interplay of porosity, surface accessibility, light absorption, and charge-carrier dynamics rather than from band-gap modification alone.

The superior performance of TA0.5–TiO_2_ demonstrates that TA can regulate the TiO_2_ framework without conventional elemental doping, providing a sustainable molecular approach to tuning its structural and textural properties. To the best of our knowledge, this study provides the first demonstration of TA as a bio-based structure-directing agent for the sol–gel synthesis of TiO_2_.

Future studies should clarify the mechanistic links between TA-induced structural modifications, charge-carrier behavior, and reactive species generation, while evaluating catalyst stability and performance in complex aqueous matrices. Extending this approach to other semiconductor systems and heterostructures could further advance the use of bio-derived molecular structure-directing agents in photocatalyst design.

## Supplementary Material



## Notes

Data supporting the linear BET plots obtained from the selected relative pressure range of 0.05–0.20, and a table of point of zero charge (pH_pcz_) values. (PDF)
