# Supplementary material for: Tannic Acid-Directed Synthesis of Mesoporous Anatase TiO2: Linking Structural, Surface, and Thermodynamic Properties to Photocatalytic Performance
Source: Langmuir. 2026 Sep 7;42(37):27448–63. doi: 10.1021/acs.langmuir.6c03788 (PMC13602923; doi:10.1021/acs.langmuir.6c03788)
Supplement: Supplementary file 1 [file la6c03788_si_001.pdf]

Supporting Information

Tannic Acid-Directed Synthesis of Mesoporous Anatase TiO_2: Linking Structural, Surface, and Thermodynamic Properties to Photocatalytic Performance

Stefani Pelloso de Azevedo Gobetti, Hugo Henrique Carline de Lima, Emerson Marcelo Girotto, Marcos Rogério Guilherme,* Andrelson Wellington Rinaldi*

Rinaldi Research Group, Department of Chemistry, State University of Maringá, 5790 Colombo Avenue, 87020-900 Maringá, PR, Brazil.

Corresponding author: awrinaldi@uem.br mrguilherme@uem.br

Contents

Supporting data for the linear BET plots obtained over the selected relative pressure range of 0.05–0.20 and a table of point of zero charge (pH_{pcz}) values.

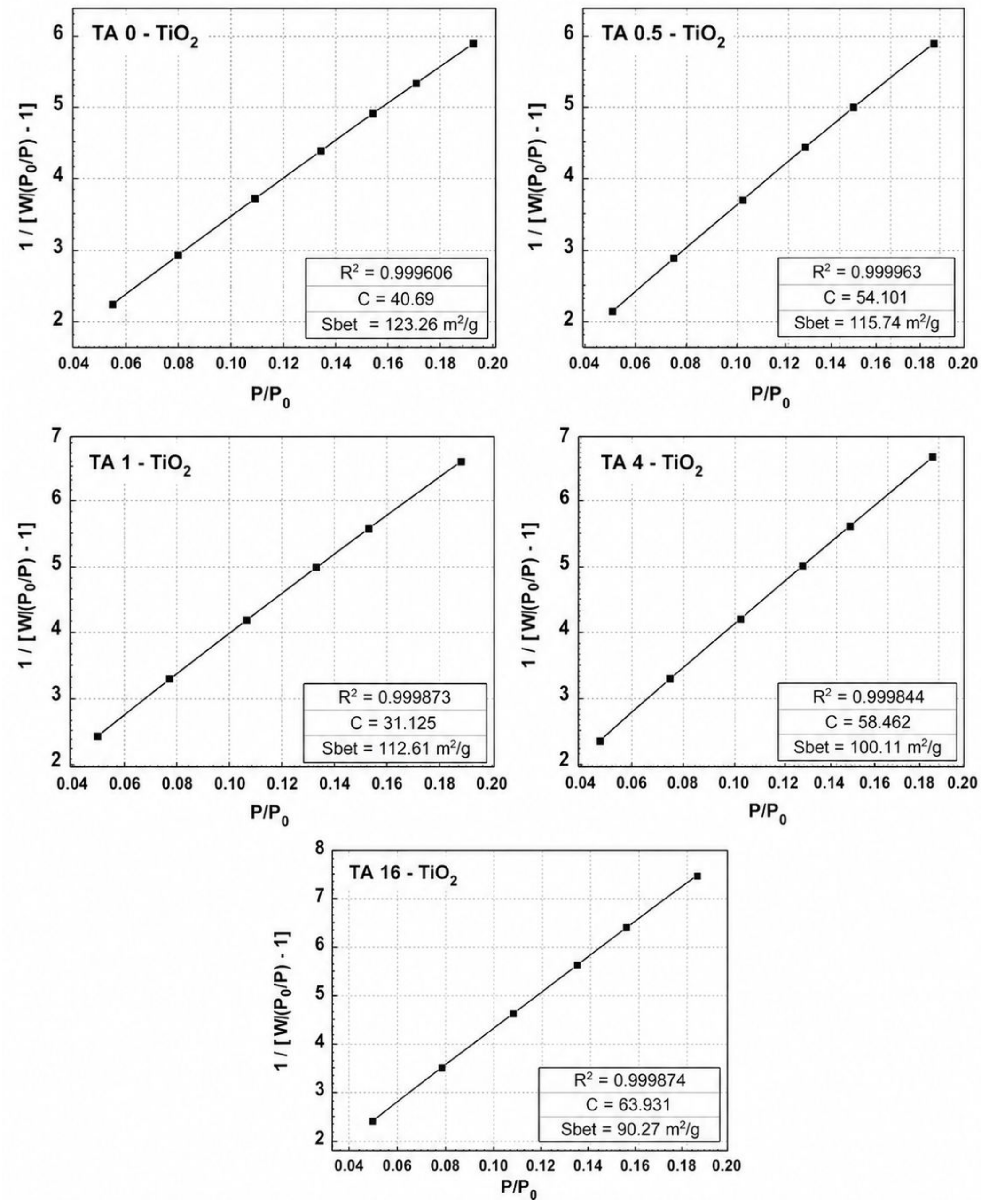

Figure S1. BET linear plots based on the selected data used for specific surface area determination in the relative pressure range of 0.05–0.20.

Table S1. Point of zero charge (pH_{pcz}) values determined for the synthesized materials.

Samples	**pH_{pcz}**
AT 0 – TiO_2	6.33
AT 0.5 – TiO_2	6.18
AT 1 – TiO_2	6.25
AT 4 – TiO_2	6.50
AT 16 – TiO_2	6.25
